# Bonwill's Method of Anæsthesia

**Published:** 1877-02

**Authors:** 


*Bonwill's Method of Anæsthesia.*—Dr. Keyser in an article on the application of this method in diseases of the Eye says· It is to my mind one of the simplest and at the same time one of the most beneficial agents in small operations about the eye that has been presented to the profession ; its application being very easy, requiring no recumbent position on the part of the patient, calling for no apparatus for its administration, and being perfectly free from any of the disagreeable effects of ether and chloroform.

To produce the proper effect the patient must open his mouth breath freely, quickly and deeply, and after a few seconds or minutes of such steady and continuous breathing, the symptoms of partial anæsthesia supervene, as is evinced by the absence of feeling in pinching or pricking with a pin. At this stage any operation should be made. The anæsthetic feeling passes almost immediately away, and the patient feels no pain in the operation if done dexterously and without hesitation.

From my experience I take pleasure in recommending its use very highly, not only to ophthalmologists, but also to the general surgeon, where minor operations are to be performed.

